# Standardization of Imaging Criteria for Detecting Macular Fibrosis in Neovascular Age-Related Macular Degeneration

**DOI:** 10.1016/j.xops.2025.101027

**Published:** 2025-12-03

**Authors:** Usha Chakravarthy, Lajos Csincsik, Kelvin Y.C. Teo, Marion R. Munk, Dilraj S. Grewal, Robyn H. Guymer, Glenn J. Jaffe, Tunde Peto, SriniVas R. Sadda, Giovanni Staurenghi, Chui M.G. Cheung, International Retinal Imaging Society Research Group, Joseph Carroll, Emil Anthony T. Say, Talisa E. de Carlo Forest, Ramtohul Prithvi, Fung Adrian, Borrelli Enrico, Bhende Muna, Querques Giuseppe, Theelen Thomas, Rao Chetan, Agarwal Aniruddha, Cicinelli Maria, Jesse J. Jung, Juan Carlos Gutiérrez Hernández, Chen Fred, Iovino Claudio, Arevalo J. Fernando, Gaudric Alain, Hartnett M. Elizabeth, Valckenberg Steffen, Rosenfeld Philip, Boyer David, Cheung Gemmy, Domalpally Amitha, Mano Fukutaro, Khalid Hagar, Moussa Magdy, Bacci Tommaso, Francisco J. Rodriguez, Huemer Josef, Souied Eric, Tsui Edmund, Frank G. Holz, Byon Iksoo, Ach Thomas, Dysli Chantal, Bousquet Elodie, Falavarjani Khalil, Krivosic Valérie, Colin S. Tan, Sivaprasad Sobha, Fabiani Claudia, Crincoli Emanuele, Chiosi Flavia

**Affiliations:** 12 Department of Ophthalmology and Visual Sciences, Medical College of Wisconsin, Milwaukee, Wisconsin; 13 South Carolina Retina Institute, Conway, South Carolina; 14 Sue Anschutz-Rodgers Eye Center, University of Colorado Anschutz Medical Campus, Aurora, Colorado; 15 Ophthalmology Department, Hopital Nord, Aix Marseille University, Marseille, France; 16 Westmead and Central (Save Sight Institute) Clinical Schools, Specialty of Clinical Ophthalmology and Eye Health, University of Sydney, Sydney, New South Wales, Australia; 17 Department of Ophthalmology, Faculty of Medicine and Health Sciences, Macquarie University, Sydney, New South Wales, Australia; 18 Department of Ophthalmology, Westmead Hospital, Sydney, New South Wales, Australia; 19 Department of Surgical Sciences, University of Turin, Turin, Italy; 20 Department of Ophthalmology, “City of Health and Science” Hospital, Turin, Italy; 21 Shri Bhagwan Mahavir Vitreoretinal Services, Medical Research Foundation, Chennai, India; 22 UniSR – IRCCS San Raffaele Scientific Institute, Milan, Italy; 23 Department of Ophthalmology, Radboud University Medical Center (Radboudumc), Nijmegen, The Netherlands; 24 Shri Bhagwan Mahavir Vitreoretinal Services, Sankara Nethralaya, Chennai, India; 25 The Eye Institute, Cleveland Clinic Abu Dhabi, Abu Dhabi, United Arab Emirates; 26 Cleveland Clinic Lerner College of Medicine, Cleveland, Ohio; 27 School of Medicine, Vita-Salute San Raffaele University, Milan, Italy; 28 Department of Ophthalmology, IRCCS San Raffaele Scientific Institute, Milan, Italy; 29 East Bay Retina Consultants, Inc, Oakland, California; 30 Department of Ophthalmology, University of California, San Francisco, San Francisco, California; 31 Universidad de Costa Rica, Caja Costarricense del Seguro Social, Hospital Clinica Biblica, San Pedro, Costa Rica; 32 Centre of Ophthalmology and Visual Science (incorporating Lions Eye Institute), The University of Western Australia, Perth, Western Australia, Australia; 33 Ophthalmology, Department of Surgery, University of Melbourne, East Melbourne, Victoria, Australia; 34 Department of Ophthalmology, Royal Perth Hospital, Perth, Western Australia, Australia; 35 Royal Victorian Eye and Ear Hospital, East Melbourne, Victoria, Australia; 36 Eye Clinic, Multidisciplinary Department of Medical, Surgical and Dental Sciences, University of Campania Luigi Vanvitelli, Naples, Italy; 37 Wilmer Eye Institute, Johns Hopkins University, Baltimore, Maryland; 38 Centre d’Imagerie et Laser, Hopital Lariboisière AP-HP, Université Paris Cité, Paris, France; 39 Byers Eye Institute of Stanford University, Palo Alto, California; 40 John A. Moran Eye Center, University of Utah, Salt Lake City, Utah; 41 Department of Ophthalmology, University of Bonn, Bonn, Germany; 42 Bascom Palmer Eye Institute, University of Miami Miller School of Medicine, Miami, South Florida; 43 Partner Retina Vitreous Associates, Los Angeles, California; 44 University of Southern California/Keck School of Medicine, Los Angeles, California; 45 Singapore National Eye Center, Singapore, Singapore; 46 Wisconsin Reading Center, University of Wisconsin, Madison, Wisconsin; 47 Department of Ophthalmology, Kindai University Faculty of Medicine, Osakasayama, Japan; 48 NIHR Moorfields Biomedical Research Centre, Moorfields Eye Hospital, London, United Kingdom; 49 Ophthalmology Department, Tanta University, Tanta, Egypt; 50 Tanta University, Tanta, Egypt; 51 Ophthalmology Unit, Department of Medicine, Surgery and Neuroscience, Siena University Hospital, University of Siena, Siena, Italy; 52 Fundonal, Escuela de Medicina y Ciencias de la Salud, Universidad del Rosario, Bogota, Colombia; 53 Moorfields Eye Hospital NHS Foundation Trust, London, United Kingdom; 54 Department of Ophthalmology and Optometry, Kepler University Hospital, Johannes Kepler University, Linz, Austria; 55 University Paris-Est Creteil, Créteil, France; 56 UCLA Stein Eye Institute, David Geffen School of Medicine at UCLA, Los Angeles, California; 57 University of Bonn, Bonn, Germany; 58 Pusan National University Hospital, Pusan National University School of Medicine, Pusan, South Korea; 59 Department of Ophthalmology, University Hospital Bonn, Bonn, Germany; 60 Department of Ophthalmology, University Hospital Bern, Bern, Switzerland; 61 Department of Ophthalmology, Lariboisière Hospital, Assistance Publique-Hôpitaux de Paris, University of Paris Cité, Paris, France; 62 Eye Research Center, The Five Senses Health Institute, Iran University of Medical Sciences, Tehran, Iran; 63 Department of Ophthalmology, Lariboisière Hospital, Assistance Publique-Hôpitaux de Paris, University of Paris Cité, Paris, France; 64 National Healthcare Group Eye Institute, Tan Tock Seng Hospital, Singapore, Singapore; 65 NIHR Moorfields Biomedical Research Centre, Moorfields Eye Hospital, London, United Kingdom; 66 Ophthalmology Unit, Department of Medicine, Surgery and Neurosciences, University of Siena and Azienda Ospedaliero-Universitaria Senese [European Reference Network (ERN) for Rare Immunodeficiency, Autoinflammatory, and Autoimmune Diseases (RITA) Center], Policlinico “Le Scotte,”, Siena, Italy; 67 Policlinico Universitario Agostino Gemelli, Rome, Italy; 68 A.O.R.N. dei Colli – Monaldi Hospital, Napoli, Italy; 1 Centre for Public Health, Faculty of Medicine and Health Sciences, Queen’s University Belfast, Belfast, United Kingdom; 2 Singapore Eye Research Institute, Singapore National Eye Centre, Singapore, Singapore; 3 Duke-NUS Medical School, National University of Singapore, Singapore, Singapore; 4 Augenarzt-Praxisgemeinschaft Gutblick AG, Pfäffikon, Switzerland; 5 Department of Ophthalmology, Inselspital, University Hospital Bern, Bern, Switzerland; 6 Department of Ophthalmology, Feinberg School of Medicine, Northwestern University, Chicago, Illinois; 7 Department of Ophthalmology, Duke University Medical Center, Durham, North Carolina; 8 Centre for Eye Research Australia, Royal Victorian Eye and Ear Hospital, East Melbourne, Victoria, Australia; 9 Ophthalmology, Department of Surgery, The University of Melbourne, Melbourne, Victoria, Australia; 10 Doheny Eye Institute, David Geffen School of Medicine, University of California, Los Angeles, Los Angeles, California; 11 Eye Clinic, Department of Biomedical and Clinical Sciences “Luigi Sacco”, University of Milan, Milan, Italy

**Keywords:** Age-related macular degeneration, Anti-VEGF, Consensus, Fibrosis, International Retina Imaging Society

## Abstract

**Purpose:**

To evaluate conventional imaging modalities for detecting fibrosis in neovascular age-related macular degeneration (nAMD) and to develop a standardized diagnostic workflow.

**Design:**

Systematic discussion and grading exercise assessing multiple imaging modalities.

**Participants:**

Retina specialists from the International Fibrosis Consensus workgroup and members of the International Retinal Imaging Society.

**Methods:**

An international panel assessed the advantages and limitations of 5 imaging modalities—color fundus photography (CFP), fluorescein angiography (FA), spectral domain OCT (SD-OCT), near-infrared reflectance, and fundus autofluorescence—for detecting fibrosis in nAMD. A structured debate was followed by 2 online, masked image grading surveys. Sensitivity, specificity, and predictive accuracy of each modality, alone and in combination, were determined. Intergrader agreement was calculated. Imaging features were also correlated with histology in a nonhuman primate laser model. Based on consensus discussions at 2 in-person meetings and survey results, a 2-step diagnostic approach using SD-OCT as the primary modality was proposed.

**Main Outcome Measures:**

Recommendation for a standardized approach for diagnosing fibrosis in eyes with nAMD.

**Results:**

Among the 5 modalities, SD-OCT was considered essential by all workgroup members. Hyperreflective material on OCT was unanimously identified as a key indicator of fibrosis. However, its limited specificity was acknowledged. In 2 masked grading exercises, SD-OCT showed the highest sensitivity (0.88 and 0.84) but only moderate specificity (0.56 and 0.57). The area under the curve (AUC) for SD-OCT was 0.72 and 0.70. A 2-step strategy combining SD-OCT with CFP or FA improved diagnostic accuracy. Hyperreflective material was defined as material with reflectivity equal to or greater than normal retinal pigment epithelium (RPE), well-defined margins, RPE disruption, and a laminated appearance. Corresponding CFP findings included well-defined yellow/white/gray subretinal lesions, and FA findings included early blocked fluorescence and late staining. This 2-step approach increased AUC to 0.85, with sensitivity of 0.83 and specificity of 0.87.

**Conclusions:**

The study establishes a 2-step approach using OCT as the primary modality in clinical studies for the detection of fibrosis.

**Financial Disclosure(s):**

Proprietary or commercial disclosure may be found in the Footnotes and Disclosures at the end of this article.

Fibrosis is an important cause of vision loss in eyes with neovascular age-related macular degeneration (nAMD).^1^, ^2^, ^3^ After the introduction of treatments that block anti-VEGF, systematic reviews show heterogeneity in reported event rates for the incidence of fibrosis; some indicate impressive reductions compared with prior decades, whereas others suggest more modest effects.^4^^,^^5^ Nonetheless, the presence of fibrosis at presentation or new onset of fibrotic change in the macular tissues during anti-VEGF therapy is a finding that is associated with worse visual outcomes compared with eyes without macular fibrosis.^6^, ^7^, ^8^, ^9^

In eyes managed with anti-VEGF therapy, the incidence of fibrosis increases with duration of follow-up, even when retreatment intervals have been optimal.^6^, ^7^, ^8^, ^9^ These data suggest that at least in a subset of cases, fibrosis develops despite current therapeutics as presently used in clinical practice. Fibrosis is more likely to occur in type 2 macular neovascularization, larger lesions at treatment initiation and in the presence of high-risk features such as subretinal blood and hyperreflective material (HRM) on spectral domain OCT (SD-OCT) at presentation.^10^, ^11^, ^12^, ^13^, ^14^, ^15^ In 1 study with long-term follow-up, nearly half of eyes without fibrosis at baseline undergoing ongoing anti-VEGF treatment developed fibrosis by 8.3 years.^16^

With increasing efficacy and durability of therapies that control exudation, there has been renewed interest in documenting the onset, extent, and severity of fibrosis as its presence is strongly associated with decreased function despite inactive neovascularization.^6^^,^^9^ Notably, disease activity recurrence and fluctuating retinal thickness have been associated with higher fibrosis incidence regardless of number of injections received.^12^^,^^16^^,^^17^ In addition to VEGF blockade to treat nAMD, there is interest to develop agents that act by a different mechanism of action and that could inhibit fibrosis.^17^^,^^18^ Furthermore, agents with antifibrotic properties are currently being evaluated in early phase clinical trials creating urgent need for harmonized methods to detect fibrosis and to assess its the severity. There is currently no consensus on the type of imaging required or how features detected by the different imaging modalities should be used to diagnose and quantify fibrosis. However, there is an urgent need to develop trial end points for studies evaluating agents with antifibrotic properties. Although fluorescein angiography (FA) and color fundus photographs (CFPs) have been traditionally used, such as in the Comparison of Age-related Macular Degeneration Treatments Trials (CATT) study,^13^^,^^19^ some authors have proposed the use of SD-OCT as the primary modality for evaluating fibrosis.^20^ Indeed, a recent study comparing imaging modalities demonstrated significant differences in both prevalent and incident fibrosis depending upon the imaging modality used.^21^

The foregoing emphasizes an urgent need for consensus methodology in the evaluation of fibrosis, with regard to imaging modality, definition, timing of reporting, and anatomical localization with respect to foveal involvement and tissue layers affected. This report summarizes the findings from the International Fibrosis Consensus (IFC) workgroup that was formed to provide recommendations on the above. As with other recent consensus programs,^22^^,^^23^ this group was managed through International Retinal Imaging Society (IntRIS), which also provides an opportunity for the recommendations from consensus groups to be tested by a broader group of international experts. The IFC workgroup comprised retina specialists and representatives from 5 reading centers (RCs), highly experienced in nAMD trials.

## Methods

An international group of retina experts in AMD with RC experience was assembled as a part of the IFC workgroup. This multi-phase comprehensive initiative integrated clinical image grading, validation methodology, and supporting preclinical investigations.

### Deliberations on Imaging Modalities (Phase I)

Before the first meeting, workgroup members engaged in a premeeting survey that captured members’ ranking of 5 imaging modalities in the order of usefulness to detect fibrosis (CFP, FA, SD-OCT, near-infrared reflectance, and fundus autofluorescence) and their definition of fibrosis by modality. The survey also asked about preferred timing of assessment to capture onset of fibrosis in a potential clinical as well as a trial setting.

### Image Grading and Validation by IFC Workgroup (Phase II)

Phase II evaluated the performance of individual and combinations of imaging modalities reflecting recommendations from phase I. Based on the recommendation from phase I, this exercise only included CFP, FA, and SD-OCT. Images from the Early Detection of Neovascular AMD (EDNA) study were used for this exercise (see following paragraphs for details of the EDNA study). Specifically, 10 eyes representing a broad range of examples with and without fibrosis in the setting of macular neovascularization of different types at the 18-month visit of the EDNA study were selected. The gold standard was the grade assigned by a senior grader (L.C.) and adjudicated by an experienced retina specialist (C.M.G.C.). Intergrader variability was assessed as well.

Workgroup members used a web-based platform to evaluate each individual imaging modality, namely CFP alone, FA alone, and SD-OCT alone, for the presence/absence of fibrosis. To minimize bias arising from participants recall of findings from one imaging modality influencing the grading of a different imaging modality, the images from any one modality was made available over a specified window of time. After an interval of around 3 weeks, the next set of images from a different imaging modality was then made available for grading and in a different order to the first. This process was repeated for the third imaging modality. The workgroup received access to the next imaging modality only when the full set from the prior image modality had been graded and submitted. It was therefore impossible to compare or use the other image modalities for cross checking. Performance of individual imaging modality and agreement between each was tested against the gold standard for detection of fibrosis.

### Image Grading and Validation by IntRIS Members (Phase III)

The grading exercise was repeated with a broader workgroup, which included members of the IntRIS using the same methodology as phase II. In addition, IntRIS members were also invited to indicate their level of agreement with 4 recommendations related to imaging modality and definition for fibrosis. Forty-two members responded to the survey.

For the clinical studies, images and clinical data used were extracted from a repository obtained from the EDNA study.^24^ The study design and results of EDNA have been published in detail.^24^ Early Detection of Neovascular AMD is a diagnostic accuracy study that enrolled participants across 24 retina clinics in the United Kingdom to determine the optimum technology to detect the onset of neovascularization in high-risk eyes (study eyes) of participants with confirmed nAMD in the first eye (fellow eyes) at initial presentation. All participants gave informed consent, and the study was conducted in accordance with the tenets of Declaration of Helsinki and approved by the office for Research Ethics Committees Northern Ireland (study ID 14NI/1120). Images from the EDNA data repository of 552 participants from the baseline nAMD eyes that were on treatment with anti-VEGF agents were used for the survey. Ten cases covering a range to neovascularization subtypes were selected sequentially from the database at the 18-month visit as this was a mandated study visit at when CFP, FA, and SD-OCT images were obtained.^21^ An online database using Microsoft OneDrive was established to facilitate image sharing for all 3 grading phases, with access provided to the designated graders. OCT scans were exported from the EDNA database via Heyex software (v1.10.4.0) using the “Export as AVI” function with the Microsoft MPEG-4 Video Codec V2. To ensure compatibility with both Windows and Mac, the resulting AVI files were converted to MP4 format. All scans were fovea-centered volume scans and acquired in high-speed mode (A-scan count: 768) using the Spectralis OCT system (Heidelberg Engineering), consisting of either 31 or 61 B scans covering a 30° × 25° area. In addition to the OCT scans, a fovea-centered early phase and late-phase FA frame were exported from the EDNA database in tagged image file format using the XML export function within the same platform. Color fundus photographs were exported in JPG format from the EDNA database via Oculab software. Color fundus photographs were acquired using a Canon imaging system. Online questionnaires and grading forms for all 3 phases were created using Google Forms, and the recorded data were stored as comma-separated file files on Google Drive before analysis.

### Statistical Analysis

Based on phase II and phase III responses, performance of each modality to detect fibrosis compared with the gold standard was assessed by determining sensitivity, specificity, and predictive accuracy (area under the curve [AUC] and 95% confidence intervals [CIs]). Intergrader agreement for each modality was calculated using Fleiss kappa. Kappa levels were interpreted as follows; between 0.0 and 0.2 agreement was low, fair if between 0.21 and 0.4, moderate if between 0.41 and 0.6, and good if >0.61, respectively. In addition, combinations of imaging modalities were analyzed based on responses from the phase III grading exercise to assess whether multimodal integration improved diagnostic performance, with AUC computed for each combination. To quantify intergrader variability beyond Fleiss kappa, we fitted linear mixed-effects models for each imaging modality. For each model, the grading score was treated as the dependent variable, with random intercepts for subject and grader to partition variance into components attributable to true subject differences, grader differences, and residual variability. Subject differences refer to variability due to differences between true biological signal, grader differences refers to intergrader variance, and residual unexplained variance may be due to factors such as within-grader or random noise. Variance components were extracted to calculate the proportion of total variance attributable to graders, representing between-grader variability.

All analysis and data visualization were carried out in R (version 4.3.2, R Foundation for Statistical Computing).

## Results

### Results of Phase I Deliberations

#### Deliberations on Individual Imaging Modalities and Definitions

Among the 5 imaging modalities considered (CFP, FA, SD-OCT, near-infrared reflectance, and fundus autofluorescence), SD-OCT was considered as essential by 10 of 10 (100%) of workgroup members (Table 1). On SD-OCT, the presence of HRM was unanimously proposed and considered to be essential for diagnosing fibrosis. Hyperreflective material internal to the retinal pigment epithelium (RPE; i.e., subretinal location) was unanimously accepted as relevant to fibrosis, but there was debate as to whether HRM external to the RPE (sub-RPE) should be considered. Advantages of SD-OCT as a diagnostic modality include its wide availability, noninvasiveness and that it can be repeated at every visit. However, limited specificity of HRM as a diagnostic criterion for detecting fibrosis is an important limiting factor in the use of SD-OCT as the sole imaging modality. At this stage of the exercise, the workgroup suggested the inclusion of additional textural characteristics to further refine SD-OCT features for diagnosing fibrosis. Two features considered were the level of reflectivity and the margins of the HRM. Apart from fibrosis, other lesions associated with HRM on SD-OCT in the nAMD setting include blood, fibrinous exudate, and blood vessels. Hyperreflective material associated with fibrosis is characterized by high reflectivity that is comparable to that of the RPE. In contrast, HRM associated with other lesion components rarely exhibit such high reflectivity. With regard to the margins of HRM, well-defined margin was considered helpful in differentiating fibrosis from other lesion components. Presence of band-like lamination was also considered helpful in differentiating HRM associated with fibrosis from other lesion components.

**Table 1:** Summary of Imaging Modalities Considered for Fibrosis Detection by Consensus Workgroup Members

Modality	Role in Fibrosis Detection	Appearance	Comments	Further Considerations
SD-OCT	Essential (100%)	Reflectivity—HRM Level—can be internal or external to RPE, if RPE layer is visible	Widely available Noninvasive High sensitivity Limited specificity	Reflectivity Margin Banded/laminated Sub-RPE? Must there be RPE disruption/loss Must there be EZ disruption/loss
CFP	Essential (62%)	Color—yellow/white/gray Shape/outline—well-defined ± Level-elevated	Widely available Noninvasive Image quality more susceptible to artifacts Good for thick, subretinal fibrosis Lower sensitivity for thin fibrosis, fibrosis external to RPE May be affected by atrophic lesions	Conventional vs. pseudocolor or multicolor
FA	Optional (80%)	Early—blocked fluorescence Late—staining, (fading)	Invasive	
NIR	Not routinely used	Variable brightness		
FAF	Not routinely used	HypoAF	Can be used to exclude causes of HRM other than fibrosis that present as hyper-AF lesions (e.g., vitelliform materials or blood).	

AF = autofluorescence; CFP = color fundus photography; EZ = ellipsoid zone; FA = fluorescein angiography; FAF = fundus autofluorescence; HRM = hyperreflective material; NIR = near-infrared reflectance; RPE = retinal pigment epithelium; SD-OCT = spectral domain OCT.

**Figure 1:**
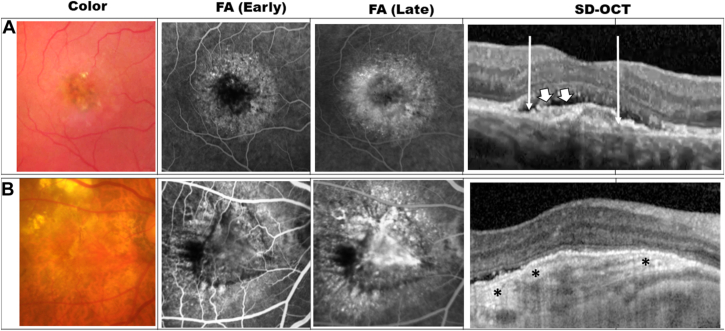
Multimodal imaging examples of fibrosis lesion in the setting of neovascular age-related macular degeneration. Each row shows multimodal imaging comprising color fundus photography, early phase fluorescein angiography (FA), late-phase FA, and spectral domain OCT (SD-OCT). (**A**) Fibrosis internal to the retinal pigment epithelium (RPE), (**B**) fibrosis external to the RPE. Typical fibrosis exhibited a combination of highly hyperreflective material (white block arrows) with well-defined margins and lamination (∗), with disruption of overlying RPE (extent of RPE disruption delineated by white arrows).

Color fundus photography was stated as essential by 5 of 8 (62%) of workgroup members with the following features needing to be assessed: color of lesion, outline and level included in the consideration of presence of fibrosis. The workgroup recommended that the definition for fibrosis on CFP should be an elevated, yellow/white/gray lesion with a well-defined border. The workgroup noted that the appearance of fibrosis on CFP can be affected by its location whether internal or external to the RPE, the thickness of fibrotic tissue, and the presence of RPE atrophy. Fibrosis that is subretinal, that is, internal to the RPE, is more readily detected on CFP compared with fibrosis external to the RPE. Thicker fibrotic tissue is more readily detected compared with a thin layer. Presence of RPE atrophy appears as a depigmented area on CFP and in the absence of stereoscopic information, may be confused with thin fibrosis. Figure 1 shows examples of multimodal imaging findings in eyes with fibrosis. Multimodal imaging findings of lesions that may be confused with fibrosis based on a single imaging modality are shown in Figure S2 (available at www.ophthalmologyscience.org).

Fluorescein angiography was considered as an optional modality for fibrosis detection by 8 of 10 (80%) workgroup members. The workgroup recommended the definition on FA to include early blocked fluorescence and late staining of the lesion. However, differentiating leakage from staining may be challenging and could contribute to false-positive grading for fibrosis. On the other hand, in lesions with areas of mixed active neovascularization and fibrosis, leakage may predominate and mask the detection of fibrosis. A detailed description of imaging appearance considered important in fibrosis by each workgroup member is included in Table S2 (available at www.ophthalmologyscience.org).

Fundus autofluorescence and near-infrared reflectance were not used by any of the workgroup members routinely to detect fibrosis and not included in the recommendation. However, fundus autofluorescence can be used to exclude other causes of HRM that may present as hyper-AF lesions (e.g., vitelliform materials or altered blood).

#### Deliberations on Preferred Timing of Assessment to Capture Onset of Fibrosis

The workgroup agreed that eyes with HRM at the acute exudative phase of nAMD are at high risk of developing fibrosis. Although HRM can completely resolve with treatment using anti-VEGF in some eyes, around one half continue to exhibit persistence that may be considered to be fibrosis,^3^^,^^20^ although robust histological proof of this transition is lacking. Therefore the evolution and transition patterns of HRM from the treatment-naïve state into fibrosis over time requires detailed longitudinal study. For the purpose of detecting incident fibrosis among eyes without fibrosis at baseline, the workgroup agreed that the onset of fibrosis after treatment initiation could be observed as early as at 1 month on OCT when transient HRM has resolved. However, the majority of workgroup members preferred a time point of 6 to 12 months as the optimal point in time to evaluate fibrosis after treatment initiation. The workgroup noted that in the treatment-naïve phase, HRM on SD-OCT can be due to whole blood, fibrinous exudate, and blood vessels, all of which are important constituents as they facilitate subsequent fibrosis development. In the immediate aftermath of initiating treatment, HRM due to these materials can resolve entirely. However, if HRM fails to resolve or recurs, reflecting inadequate control of disease activity within the neovascular lesion, the risk of fibrosis development is increased. Therefore, a time point of ≥6 months post treatment initiation is recommended to detect persistent HRM that can be considered to be fibrosis, as any resolvable hyperreflective constituents will most likely have disappeared by this juncture.

### Results of the Phase II Grading by IFC Workgroup

Sensitivity, specificity and AUC were computed against the gold standard comparator by the 7 experts (Table 3). Sensitivity (95% CI) was highest for SD-OCT (0.88 [0.66–0.97]), followed by FA (0.85 [0.60–0.94]) and CFP (0.69 [0.35–0.96]). However, specificity was highest for CFP (0.70 [0.36–1.00]), low for FA (0.38 [0.09–0.67]), and moderate for SD-OCT (0.56 [0.24–0.88]). The overall AUC (95% CI) was 0.72 (0.62–0.82) (SD-OCT), 0.69 (0.59–0.79) (CFP), and 0.62 (0.52–0.72) (FA). Interobserver agreement assessed with Fleiss kappa was 0.54 (CFP), 0.32 (FA), and 0.29 (SD-OCT). Variance component analysis showed that most of the variability in grading scores was attributable to differences between subjects, with relatively small contributions from graders. For color fundus images, between-subject variance accounted for 58.9% of the total variance, between-grader variance for 2.2%, and residual variance for 38.9%. For FA, between-subject variance was 30.2%, grader variance 5.2%, and residual variance 64.6%. For OCT, between-subject variance accounted for 23.6%, grader variance 3.5%, and residual variance 72.9%.

**Table 3:** Performance of Individual and Combination Imaging Modalities Compared with Gold Standard Grading for Detecting Fibrosis in Eyes with Neovascular Age-Related Macular Degeneration

Modality	AUC	Sensitivity	Specificity
Phase I: Expert workgroup (n = 7)			
CFP	0.69 (0.59–0.79)	0.69 (0.35–0.96)	0.70 (0.36–1.00)
FFA	0.62 (0.52–0.72)	0.85 (0.60–0.94)	0.38 (0.09- 0.67)
OCT	0.72 (0.62–0.82)	0.88 (0.66–0.97)	0.56 (0.24–0.88)
Phase II: IntRIS members (n = 42)			
CFP	0.77 (0.73–0.81)	0.83 (0.78–0.88)	0.71 (0.66–0.76)
FFA	0.65 (0.61–0.70)	0.76 (0.69–0.81)	0.55 (0.50–0.61)
OCT	0.70 (0.66–0.74)	0.84 (0.78–0.89)	0.57 (0.51–0.63)
Proposed stepped approach			
OCT+ CFP or FFA	0.85 (0.82–0.89)	0.83 (0.77–0.89)	0.87 (0.83–0.91)

AUC = area under the curve; CFP = color fundus photography; FAF = fundus autofluorescence; FFA = fundus fluorescein angiography; IntRIS = International Retinal Imaging Society.

### Results of the Phase III Grading by IntRIS Members and Survey on 4 Consensus Statements

A summary of phase I and II findings was presented at the IntRIS annual meeting in June 2024 and members were invited to participate in the phase III grading exercise to assess the group experience within a wider cohort of retina and imaging specialists. Forty-two of the 128 invited IntRIS members with clinical background (i.e., excluded PhDs and bioengineers) participated. Findings from phase III grading were similar to that in phase II (Table 3) and confirmed prior sensitivity and performance assessments of respective image modalities: individual modalities achieved AUC (95% CI) of 0.77 (0.73–0.81) (CFP), 0.70 (0.66–0.74) (SD-OCT), and 0.65 (0.61–0.70) (FA). Sensitivity (95% CI) of individual modalities was highest for SD-OCT (0.84 [0.78–0.89]), followed by CFP (0.83 [0.78–0.88]) and lowest for FA (0.76 [0.69–0.81]). Specificity [95% CI] was highest for CFP (0.71 [0.66–0.76]), followed by SD-OCT (0.57 [0.51–0.63]) and lowest for FA (0.55 [0.50–0.61]).

Applying the 2-step, multimodal strategy based on SD-OCT + CFP or FA increased the computed AUC (95% CI) to 0.85 (0.82–0.89), with a sensitivity of 0.83 (0.77–0.89) and specificity of 0.87 (0.83–0.91).

International Retinal Imaging Society members were also asked to vote to indicate their level of agreement with the following 4 recommendations proposed based on the deliberations in phase I. Responses showed high level of agreement (based on >80% indicating strongly agree or agree) for 3 statements with 90%, 95%, and 83% respectively for statements 1 to 3.

A fourth statement returned 67% responses as strongly agree or agree.

1. Multimodal imaging is recommended for the detection of fibrosis (38/42, 90%).
2. The definition of fibrosis on SD-OCT: HRM appearing as either homogeneously hyperreflective or laminated/band-like areas under the retina (40/42, 95%).
3. The definition of fibrosis on CFP: the presence of well-defined yellow/white/gray lesion under the retina (35/42, 83%).
4. The definition of fibrosis on FA: early blocked fluorescence and late staining (28/42, 67%).

### Final Recommendations

A 2-step approach for diagnosing fibrosis in eyes with nAMD was recommended. Fibrosis can be considered as definitely present in the presence of 1 + 2a/2b as follows:

1. SD-OCT demonstrating:
  a. presence of highly HRM (defined as having reflectivity level similar to or greater than that of the normal RPE),
  b. well-defined margins,
  c. evidence of RPE disruption, and
  d. laminated/banded appearance
2. a. Color fundus photo demonstrating: well-defined yellow/white/gray subretinal lesion in the area corresponding to HRM, and/or
  b. Fluorescein angiography: presence of early blocked fluorescence and late staining in the area corresponding to HRM.

## Discussion

In this report, the IFC workgroup evaluated several imaging modalities used to detect fibrosis and assessed their potential role for clinical studies and interventional trials in which fibrosis development is an anatomical end point. In this report we confirmed the high sensitivity and limited specificity of OCT and propose a strategy to improve the specificity of OCT by including textural characteristics. In particular, high hyperreflectivity and well-defined margins in addition to the presence of HRM can help to differentiate fibrosis from other lesion components. We further recommend a multimodal approach that can be adopted in future clinical studies and trials (Table 5). In the validation exercise, which compared the sensitivity and specificity of detection by a wider group of retinal practitioners and imaging experts against the gold standard grading, the proposed 2-step strategy achieved a higher AUC than any individual modality.

**Table 5:** Suggested Framework for Reading Centers Evaluating Fibrosis

	Recommendations	Suggested Framework (Can Be Modified by Individual Reading Center in Discussion with Sponsor and/or Principal Investigator)
1	Minimum image set	• Mandatory: SD-OCT volume scan • Mandatory: Color fundus photo • Conditional: Fluorescein angiography early and late frames
2	Adjudication rules (see Fig 1 for sample images)	Step 1: SD-OCT 1 HRM present a HRM reflectivity comparable to RPE b HRM margins well-defined c HRM presence of band-like lamination 2 RPE layer disruption or loss Step 2a: If yes to 1 Well-defined yellow/white/gray subretinal lesion corresponding to HRM, or Step 2b: If yes to 1 Early blocked fluorescence and late staining corresponding to HRM Adjudication procedures When intergrader agreement falls below thresholds (see *Discussion* section), arbitration of discrepant cases independently by a senior grader, documenting reasoning and providing specific supporting image features. If disagreement persists, consensus meeting to involve all graders after structured evaluation protocols. Final adjudicated results become gold standards for analysis.
3	Training	Each reading center will establish its own Quality Control (QC) Framework Training of graders should be conducted in a structured manner with prespecified Grader Certification requirements. All graders must complete initial training comprising didactic instruction, followed by supervised interpretation of ≥200 images. Certification involves reading a standardized set of 100 images with known diagnoses, achieving minimum agreement levels of *K* ≥ 0.80 for primary diagnoses and *K* ≥ 0.70 for secondary features.
4	Quality control	Follow established protocol per individual reading center, for example: -Duplicate grading: Reading centers may choose the number of study images that are graded a second time. This can vary from 1 in 20 to 1 in 5 depending on the protocol and sample size. Study images are re-entered into the grading queue for assessment using stratified random sampling. This would allow both intra- and intergrader reproducibility to be evaluated. The percentage for duplicate grading is determined by the sponsor/principal investigator at the start of the study and should be clearly stated in the protocol. If duplicate grading is not possible the following is recommended: -Intragrader Reliability Assessment: each grader rereads a minimum of 5% of their previous assessments after a minimum 4-wk interval using systematic sampling to ensure representation across different time periods and complexity levels. -Intergrader Reliability Assessment: Images are selected using stratified random sampling across disease severity levels, image quality grades and time periods within the study. Metrics for assessing intergrader agreement include: concordance rate, Cohen kappa, weighted kappa, and intraclass correlation coefficient. Acceptable agreement thresholds can be agreed with sponsor or principal investigator at the start of the study. Examples include *K* ≥ 0.80 for primary outcomes (e.g., presence of fibrosis) and intraclass correlation ≥0.90 for quantitative measures.

HRM = hyperreflective material; RPE = retinal pigment epithelium; SD-OCT = spectral domain OCT.

One of the most important reasons for discordance in the reported frequencies of prevalent and incident fibrosis before and during anti-VEGF therapy is the differences in the definitions applied by investigators in the detection of fibrosis and also by the imaging modality used. Notably in prior decades, detection of fibrosis in the macula in eyes with nAMD was based on CFP. In addition, the dynamic changes in fluorescence patterns in the macular lesions during FA were also used to identify fibrosis. Both CFP and FA are 2-dimensional techniques providing en face information, although limited depth information can be extracted through the acquisition of stereoscopic pairs of images.^25^ The current standard of care with anti-VEGF agents that effectively contain exudation in nAMD has altered the appearance from the previously florid manifestations of macular scarring to an appearance of thin fibrosis in a high proportion of eyes over time. With the use of SD-OCT instrumentation and capture of 3-dimensional images of macular lesions, lesser degrees of scarring with less thickness or volume may be visible as sheets or mounds of HRM that lie adjacent to the outer retinal layers or found to be replacing them. However, standardized definitions of fibrosis were not developed, leading to considerable variation in the interpretation of SD-OCT images. In addition, the timing of assessment after initiation of anti-VEGF treatment and suboptimal retreatment regimens leading to inadequate control of disease activity in the selected sample have been confounders in the estimation of true incident rates of fibrosis.

With respect to SD-OCT, key advantages include high sensitivity, ease of repeated acquisition, ability to evaluate the impact on adjacent functionally important structures (e.g., RPE and ellipsoid zone), and amenability to volumetric quantification. In the clinical setting, many practitioners rely solely on OCT assessment. Our recommendations to include additional textural characteristics to HRM can improve the clinical utility of OCT in identifying fibrosis. The additional specification of the level of highly HRM (greater than or equal to normal RPE reflectivity), well-defined margins, and presence of band-like laminations aim to increase the precision of an OCT-based set of diagnostic criteria for identifying fibrosis. However, despite the inclusion of additional descriptors, SD-OCT alone still lacks specificity when compared with a multimodal approach. Hence for the purpose of clinical studies and trials, confirmation with a second en face imaging modality (CFP or FA) is recommended in the 2-step multimodal approach.

The workgroup also proposed 4 recommendations regarding the imaging modality and definitions to be used for each modality. These definitions can be adopted by RCs and retinal experts in future clinical and interventional studies testing disease activity control with longer durability agents or antifibrotic agents. Our proposal of a stepwise approach with clear definitions for each imaging modality will help to standardize and harmonize the evaluation of fibrosis as an important anatomical end point in such endeavors. Table S4 (available at www.ophthalmologyscience.org) summarizes how multimodal imaging can aide in differentiating fibrosis from blood, neovascularization, drusen, atrophy, vitelliform material, hard exudates, and fibrin.

Correlation between structural findings and functional implications is important, particularly for the evaluation of therapeutic outcomes. The presence or absence of HRM, although useful as a screening tool for fibrosis detection, has limited sensitivity. The functional impact of fibrosis is likely to also depend on whether the fibrotic material is internal or external to the RPE,^4^^,^^16^^,^^21^ the integrity of the RPE layer, and/or disruption of the photoreceptor matrix and its location in relation to the foveal center. Previous studies have demonstrated that subretinal fibrosis is a major determinant of poor visual outcomes but data on fibrosis external to the RPE is less consistent.^10^^,^^21^ Indeed, the workgroup were uncertain on the visual significance of sub-RPE fibrosis and whether this is simply an integral component of fibrovascular pigment epithelial detachment (PED), the latter having been shown to have better functional outcomes compared with eyes with a predominantly serous PED.^26^ Some workgroup members expressed the view that not all fibrovascular PEDs are fibrotic with others arguing that the term itself implies the presence of elements of fibrosis. Although fibrosis external to the RPE tends to have a less pronounced negative impact on visual function, studies have demonstrated that compared with eyes without HRM, eyes with HRM external to the RPE have worse visual function.^4^^,^^9^ Furthermore, several studies that follow eyes with fibrosis report expansion and progression of fibrosis, which is accompanied by increasing disruption of the RPE, photoreceptors, and external limiting membrane.^27^ The term disorganized retinal outer layers was proposed by Peto et al,^9^ who reported that when disorganized retinal outer layers was present, best-corrected visual acuity was worse in eyes with thicker retina, which was likely a reflection of fibrosis. The impact may also take longer to manifest as a result of the eventual damage to the RPE resulting from its physical separation from the underlying choriocapillaris. The group concluded that further work and analysis of longitudinal studies of sub-RPE fibrosis would be essential, and at present, the proposed definitions for fibrosis would best be applied to subretinal lesions. It is also worth noting that previous studies have used different definitions for fibrosis, which further compounds the heterogeneity of the results. Future studies using the proposed grading strategies can be undertaken to standardize the diagnosis of fibrosis and clarify the associations of fibrosis and outer retinal structure integrity with visual function.

Our work has several limitations. The current findings were based on images obtained 18 months after initiation of treatment and therefore aimed to define “definite” cases of fibrosis. The evolution of HRM, especially ill-defined, which could become persistent during the course treatment, has not been evaluated, and will be a topic of future studies. Future studies should also address how soon after commencing treatment incident HRM with features of fibrosis becomes manifest. Additionally, future studies are needed to evaluate not only a binary assessment but rather explore the best and most robust way to quantify fibrosis. A further limitation is the generalizability of our findings as the SD-OCT volume scans had variable density ranging from 31 to 67 B scans and was restricted to eyes imaged on the Heidelberg Spectralis instrument. Although we believe the interpretation related the appearance of fibrosis on SD-OCT should be transferrable, differences in instrument sensitivity, scan density, and averaging could affect interpretation. In addition to traditional true color cameras, the use of pseudocolor or multicolor images have not been assessed. Previous studies have reported different appearance for lesions such as PED in pseudocolor or multicolor from true color.^28^^,^^29^

In conclusion, we demonstrated that OCT-detected HRM alone lacks specificity. We recommend a 2-step approach wherein OCT serves as the primary screen and CFP or FA provides confirmatory evidence of fibrosis. These recommendations reflect the status of our current knowledge and available imaging technologies, as well as practical considerations. These recommendations will need to be used in practice and be modified as required in an iterative process. Learnings from the current study can inform future trials in terms of minimum image set required and adjudication rules. Other RCs’ adoption and experience will help to further validate and improve the current recommendations. However, these recommendations will need to be updated as new information becomes available and imaging technology advances. In addition to a binary assessment of presence of fibrosis, future developments should aim to quantify the area and volume of fibrosis, assess topographic distribution using en face strategies, and incorporate deep learning or artificial intelligence-assisted strategies.

## Data Availability

Data are available upon request from authors.

## Supplementary Data

Supplementary Table 1

3Supplementary Table 2

Figure S2 Multimodal imaging examples of differential diagnoses of fibrosis. Each row shows multimodal imaging comprising color fundus photograph (CFP), early phase fluorescein angiography (FA), late phase FA and spectral domain OCT. (**a**) Atrophy, (**b**) Blood, (**c**) Type 2 macular neovascularization, (**d**) Drusen, (**e**) exudates, (**f**) fibrin, (**g**) Type 1 MNV. Note hyperreflective material (HRM) is present in several non-fibrotic lesions but these differ from fibrosis as they do not exhibit a combination of highly- hyper-reflective material with well-defined margins and lamination, with disruption of overlying RPE.
