# Supplementary material for: Standardization of Imaging Criteria for Detecting Macular Fibrosis in Neovascular Age-Related Macular Degeneration
Source: Ophthalmol Sci. 2025 Dec 3;6(2):101027. doi: 10.1016/j.xops.2025.101027 (PMC12830330; doi:10.1016/j.xops.2025.101027)
Supplement: Supplementary Table 1 [file mmc1.pdf]

Supplementary Table 3. Workgroup members' proposed definition for fibrosis on SD-OCT, CFP and FA

	Definition	Comment
**SD-OCT**		
1	Well defined HRM, generally homogenous in reflectivity but may have bands. Can be above, involving or under RPE	Challenge in deciding where RPE is. Point to discuss and is probably a key issue in this exercise
2	subretinaL. defined as presence of either band-like or plaque-like areas of fibrosis underneath the retina due to MNV usually homogeneously hyperreflective and located subretinal, usually with loss of overlying PR layer, sometimes in vicinity of ORT or degenerative cysts. sometimes hard to differentiate from SHRM<br>SubRPE fibrosis: homogenous hyperreflectve band like or plaque like lesion udner RPE, usually some RP preserved.. often hard to differentiate from FV PED	primary image modality
3	Hyper-reflective bands which are well defined with homogeneous mostly horizontally oriented bands of reflectivity	
4	Presence of outer retinal hyperreflective material<br>Presence of lamination if sub-RPE	More confident If well defined, if ill-defined will definitely need to correlate with other modalities
5	hyperreflective' on OCT<br>with a location 'between neurosensory retina and RPE/Bruch's membrane,' 'in the subretinal or sub-RPE space/compartment' or 'at the RPE level. lesion obscures or replaces the normal reflectivity and banding of the neurosensory retina and RPE/Bruch's membrane complex	Bachmeier et al 2023
6	Brightly reflective/dense SHRM with sharp demarcation from overlying retina	
7	moderate hyperreflectiove dense tissue or multilayer hyperreflective lesion	
8	Homogenous SHRM with hyperreflectivity matched or exceeded that of RPE within the same B-scan. The SHRM of subretinal fibrosis was associated with RPE atrophy, overlying photoreceptor loss, and often choroidal hypertransmission	
9	Well defined HRM, generally homogenous in reflectivity but may have bands. Can be above, involving or under RPE	Challenge in deciding where RPE is. Point to discuss and is probably a key issue in this exercise
**CFP**		
1	Yellow well defined lesion in the absence of blood or areas with ill defined borders	Will correlate with OCT. blood and or fibrin (areas with ill defined borders) will obscure the true extent/area of fibrosis
2	yellowish/whitish subretinal either plaque like or band wide tissue , not caused by other morphological featutes such as drusen, Hard exudates, fibrin or dehemogloninized hemorrhage	assessed in combi with other image modalities

3	Well defined elevated mounds or regions of white greyish or yellow lesions	White and yellow but grey also if multicolor or if psuedophakic
4	Grayish-white subretinal material	
5	well- delineated areas of yellow-white tissue	
6	white(ish), yellow(ish) and/or gray (ish) lesion, well-circumscribed , elevation	Bachmeier et al 2023
7	Well-demarcated whitish-grayish material deep to the retina	TAP/VIP/SST
8	White Lesion	
9	Yellowish white, raised lesion with well-demarcated borders	
**FA**		
1	Early HyperF well defined borders with no or minimal late leak	Can be challenging the presence of CNV. Most useful in quiescent lesions
2	early hypofluorescent and minimally stained in the late-phase	assessed for presence of fibrotoc scar only together with OCT
3	Regions of blocked fluorescence in early frames and staining which fades in the late frames	Sometimes edges can leak FA and blur if scar is vascularized
4	Area of hyperfluorescence which does not leak (Staining) in late phase	
5	early phase hypofluorescence followed by late phase hyperfluorescence and staining	
6	blocked fluorescence and/or staining, with minimal or no leakage in late angiographic frames	Bachmeier et al 2023
7	Well-demarcated material deep to the retina which shows early blockage but extensive late staining	TAP/VIP/SST
8	Masking effect (dark)	
9	Well-demarcated hyperF or blocked F of underlying choroid primarily in late phase	
