# Supplementary material for: Standardization of Imaging Criteria for Detecting Macular Fibrosis in Neovascular Age-Related Macular Degeneration
Source: Ophthalmol Sci. 2025 Dec 3;6(2):101027. doi: 10.1016/j.xops.2025.101027 (PMC12830330; doi:10.1016/j.xops.2025.101027)
Supplement: 3Supplementary Table 2 [file mmc2.pdf]

Supplementary Table 2. Using multimodal imaging to differentiate fibrosis from other lesions

	Common	Different
**Fibrosis vs Blood (Figure 1a,b)**		
OCT	HRM present in both	1. HRM Reflectivity<br>Fibrosis: HRM highly hyperreflective<br>Blood: HRM variable hyperreflectivity<br>2. HRM Margin<br>Fibrosis: well-defined<br>Blood: Ill-defined<br>3. HRM Lamination<br>Fibrosis: may be laminated<br>Blood: no lamination
		RPE band<br>Fibrosis: partially/wholly disrupted<br>Blood: may be undisrupted
	EZ band disruption: variable in both	
CFP		Fibrosis: Elevated yellowish white lesion<br>Blood: Elevated lesion, variable color (altered blood may appear yellowish)
	Underlying choroidal vessels obscured	
FA	Early phase<br>blocked fluorescence in both	Late phase<br>Fibrosis: staining<br>Blood: masking
FAF		Fibrosis: HypoAF<br>Blood: variable (altered blood appears hyperAF)
**Fibrosis vs Type 2 MNV (Figure 1a,c)**		
OCT	HRM present	1. HRM Reflectivity<br>Fibrosis: HRM highly hyperreflective<br>Type 2 MNV: HRM variable hyperreflectivity<br>2. HRM Margin<br>Fibrosis: well-defined<br>Type 2 MNV: Ill-defined<br>3. HRM Lamination<br>Fibrosis: may be laminated<br>Type 2 MNV: no lamination
		RPE band<br>Fibrosis: partially/wholly disrupted<br>Type 2 MNV: may be undisrupted
	EZ band disruption: variable in both	
CFP		Fibrosis: Elevated yellowish white lesion<br>Type 2 MNV: Elevated Greyish lesion
	Underlying choroidal vessels obscured	
FA		1. Early phase<br>Fibrosis: blocked fluorescence<br>Type 2 MNV: lacy hyperfluorescence<br>2. Late phase<br>Fibrosis: staining<br>Type 2 MNV: leak/ stain depending on activity
FAF		Fibrosis: HypoAF<br>Type 2 MNV: variable
**Fibrosis vs Soft Drusen/ Drusenoid PED (1a, d)**		
OCT	HRM present	1. HRM Reflectivity<br>Fibrosis: HRM highly hyperreflective<br>Drusen: HRM moderate hyperreflectivity

		2. HRM Lamination<br>Fibrosis: may be laminated<br>Drusen: no lamination
		RPE band<br>Fibrosis: partially/wholly disrupted<br>Drusen: usually continuous
	EZ band disruption: variable in both	
CFP	Elevated yellowish white lesion obscuring underlying choroidal vessels	
FA	Late phase<br>Staining in both	Early phase<br>Fibrosis: blocked fluorescence<br>Drusen: no blocked fluorescence
FAF		Fibrosis: HypoAF<br>Drusen: usually iso-AF
**Fibrosis vs Atrophy (Fig 1a, e)**		
OCT		1. HRM<br>Fibrosis: present<br>Atrophy: absent<br>2. RPE band<br>Fibrosis: partially/ wholly disrupted<br>Atrophy: wholly disrupted<br>3. EZ band<br>Fibrosis: partially/ wholly disrupted<br>Atrophy: wholly disrupted
CFP		Fibrosis: Elevated yellowish white lesion obscuring underlying choroidal veins<br>Atrophy: pale lesion, Not elevated, increased visibility of underlying choroidal veins
FA		Early phase<br>Fibrosis: blocked fluorescence<br>Atrophy: window defect
FAF	HypoAF in both	
**Fibrosis vs vitelliform materials (Fig 1a, f)**		
OCT	HRM present	1. HRM Reflectivity<br>Fibrosis: HRM highly hyperreflective<br>Vitelliform material: HRM moderate hyperreflectivity<br>2. HRM Margin<br>Fibrosis: well-defined<br>Vitelliform material: Ill-defined<br>3. HRM Lamination<br>Fibrosis: may be laminated<br>Vitelliform material: no lamination
		RPE band<br>Fibrosis: partially/wholly disrupted<br>Vitelliform material: usually continuous
	EZ band disruption: variable in both	
CFP	Elevated yellowish white lesion obscuring underlying choroidal veins present in both	Fibrosis: well-defined margin<br>Vitelliform lesion: ill-defined margin
FA	Early phase<br>blocked fluorescence in both	Late phase<br>Fibrosis: staining<br>Vitelliform lesion: masking
FAF		Fibrosis: HypoAF<br>Vitelliform lesion: HyperAF
**Fibrosis vs Type 1 MNV (Fig 1a, g)**		
OCT	HRM present in both	1. HRM Reflectivity<br>Fibrosis: HRM highly hyperreflective

		Type 1 MNV: HRM moderate hyperreflectivity<br>2. HRM Margin<br>Fibrosis: well-defined<br>Type 1 MNV: Ill-defined<br>3. HRM Lamination<br>Fibrosis: may be laminated<br>Type 1 MNV: no lamination
	RPE band<br>partially/wholly disrupted in both	
	EZ band<br>Partially/ wholly disrupted in both	
FA		Early phase<br>Fibrosis: block fluorescence<br>Type 1 MNV: no hyperfluorescence<br>Late phase<br>Fibrosis: staining<br>Type 1 MNV: stippled hyperfluoresence
FAF		Fibrosis: HypoAF<br>Type 1 MNV: variable FAF
**Fibrosis vs Hard exudates (Fig 1a, h)**		
OCT	Highly hyperreflective HRM present in both	Signal attenuation<br>Fibrosis: not associated with intense signal attenuation<br>Hard exudates: attenuation of signal into the choroid<br>HRM Lamination<br>Fibrosis: may be laminated<br>Hard exudates: no lamination
	RPE band<br>partially/wholly disrupted in both	
	EZ band<br>Partially/ wholly disrupted in both	
CFP	Underlying choroidal vessels obscured in both	Fibrosis: Elevated yellowish white lesion<br>Hard exudates: Elevated pale whitish lesion
FA	Early phase<br>blocked fluorescence in both	Late phase<br>Fibrosis: staining<br>Hard exudates: masking
FAF	HypoFAF in both	
**Fibrosis vs Fibrin (Fig 1a, i)**		
	HRM present in both	1. HRM Reflectivity<br>Fibrosis: HRM highly hyperreflective<br>Fibrin: HRM moderate hyperreflectivity<br>2. HRM Margin<br>Fibrosis: well-defined<br>Fibrin: Ill-defined<br>3. HRM Lamination<br>Fibrin: may be laminated<br>Fibrin: no lamination
	RPE band<br>partially/wholly disrupted in both	
	EZ band<br>Partially/ wholly disrupted in both	
CFP	Elevated yellowish white lesion obscuring underlying choroidal vessels	Lesion Margin<br>Fibrosis: well demarcated<br>Fibrin: ill-defined margin
FA		Late phase:<br>Fibrosis: staining

		Fibrin: masking/leaking depending on activity
FAF		Fibrosis: HypoAF<br>Fibrin: Iso-AF
