# Supplementary figures and images for: Standardization of Imaging Criteria for Detecting Macular Fibrosis in Neovascular Age-Related Macular Degeneration
Source: Ophthalmol Sci. 2025 Dec 3;6(2):101027. doi: 10.1016/j.xops.2025.101027 (PMC12830330; doi:10.1016/j.xops.2025.101027)

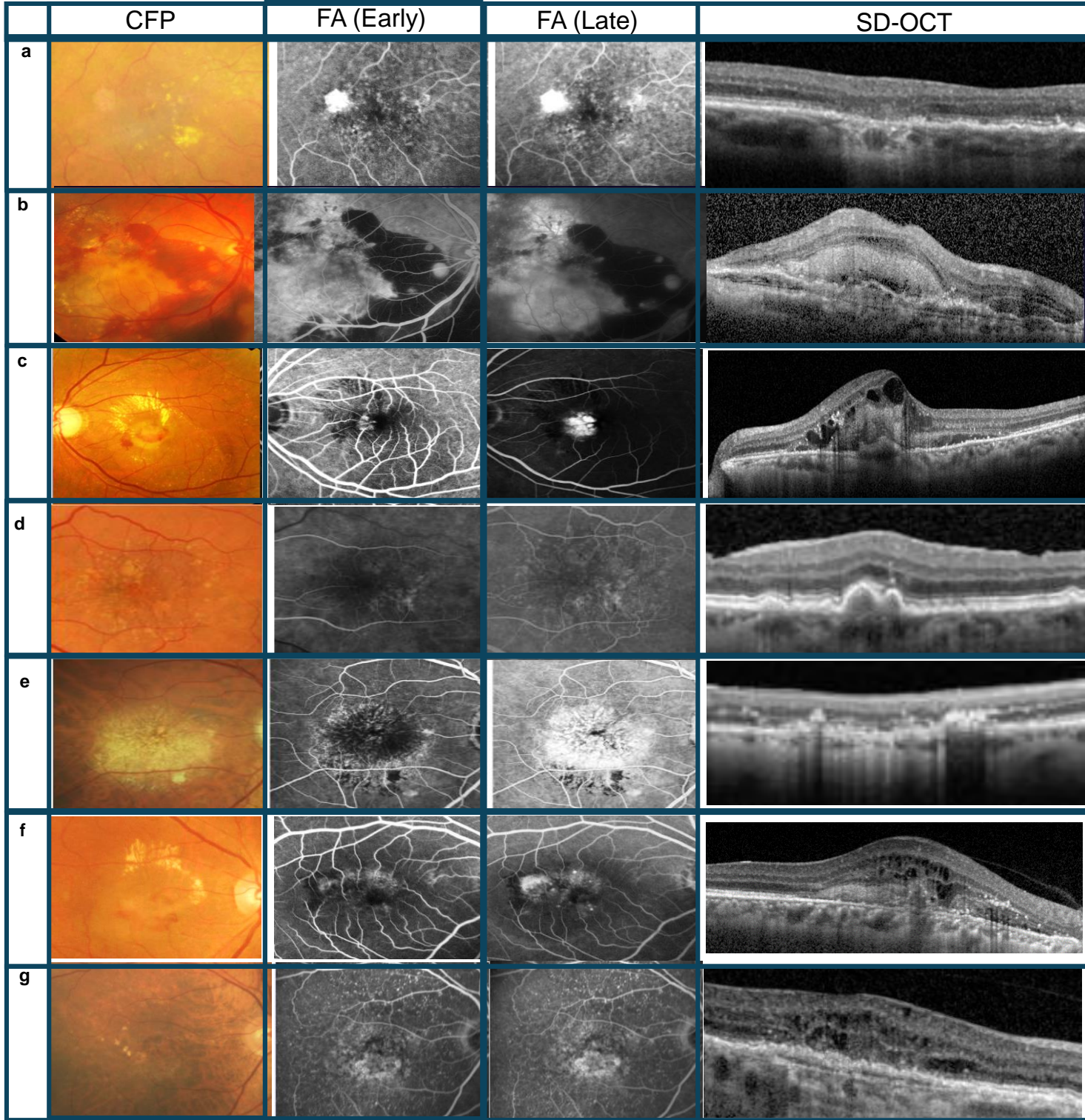

Supplement: Figure S2 — Multimodal imaging examples of differential diagnoses of fibrosis. Each row shows multimodal imaging comprising color fundus photograph (CFP), early phase fluorescein angiography (FA), late phase FA and spectral domain OCT. (a) Atrophy, (b) Blood, (c) Type 2 macular neovascularization, (d) Drusen, (e) exudates, (f) fibrin, (g) Type 1 MNV. Note hyperreflective material (HRM) is present in several non-fibrotic lesions but these differ from fibrosis as they do not exhibit a combination of highly- hyper-reflective material with well-defined margins and lamination, with disruption of overlying RPE. [file mmc3.pdf]
